# Draft genome sequence and annotation of the polyextremotolerant polyol lipid-producing fungus *Aureobasidium pullulans* NRRL 62042

**DOI:** 10.1186/s12863-024-01258-2

**Published:** 2024-08-20

**Authors:** Marie R. E. Dielentheis-Frenken, Daniel Wibberg, Lars M. Blank, Till Tiso

**Affiliations:** 1 Institute of Applied Microbiology, RWTH Aachen University, Aachen, Germany; 2 Center for Biotechnology, Universität Bielefeld, Bielefeld, Germany; 3 Computational Metagenomics, Institute of Bio- and Geosciences IBG-5, Forschungszentrum Jülich GmbH, Jülich, Germany

**Keywords:** *Aureobasidium pullulans*, Polyextremotolerant, Polyol lipid, Pullulan, Polymalic acid

## Abstract

**Objectives:**

The ascomycotic yeast-like fungus *Aureobasidium* exhibits the natural ability to synthesize several secondary metabolites, like polymalic acid, pullulan, or polyol lipids, with potential biotechnological applications. Combined with its polyextremotolerance, these properties make *Aureobasidium* a promising production host candidate. Hence, plenty of genomes of Aureobasidia have been sequenced recently. Here, we provide the annotated draft genome sequence of the polyol lipid-producing strain *A. pullulans* NRRL 62042.

**Data description:**

The genome of *A. pullulans* NRRL 62042 was sequenced using Illumina NovaSeq 6000. Genome assembly revealed a genome size of 24.2 Mb divided into 39 scaffolds with a GC content of 50.1%. Genome annotation using Genemark v4.68 and GenDBE yielded 9,596 genes.

## Objective

Strains belonging to the genus *Aureobasidium* are polyextremotolerant ascomycetes [1, 2]. This genus encompasses a diverse array of black yeasts prevalent in terrestrial and aquatic habitats across the globe. Boasting remarkable ecological versatility, *Aureobasidium* species have been isolated from soil, water, air, and even extreme environments like cold deserts and limestone caves [3–6]. While other species exist, *Aureobasidium pullulans* stands out as the most ubiquitous and well-studied member [7]. Among many beneficial traits, strains belonging to the genus *Aureobasidium* exhibit remarkable synthesis capabilities to produce a spectrum of biotechnologically interesting products, like polymalic acid, pullulan, extracellular enzymes, and polyol lipids (a.k.a. liamocins) [3, 8–10]. Additionally, *Aureobasidium* features polyextremotolerance, which is manifest by resistance towards high salt concentrations, acidic and basic conditions, and a temperature spectrum from polar to tropical conditions [11, 12]. These properties make it a potential industrial production organism. As a result, a large number of genomes have been sequenced in the past years [1, 7, 13–16].

*Aureobasidium* species are known for the production of a plethora of secondary metabolites, and two species were even named after their mainly produced secondary metabolites. While representatives of the *A. melanogenum* species often synthesize the pigment melanin [17], *A. pullulans* strains are used in industrial-scale processes to produce pullulan [18, 19]. *A. pullulans* NRRL 62042 was isolated from a leaf in Patalung, Thailand, in 2010 [20]. This strain was reported as a producer of various secondary metabolites, but is mainly known for polyol lipid production [20–22]. The polyol lipids produced by *Aureobasidium* are amphiphilic molecules and, as such, might be applicable as biosurfactants [23, 24]. Since not many strains feature the ability to produce these biosurfactants, it is of particular interest to unravel the genetic foundation and metabolic pathways underlying polyol lipid synthesis.

## Data description

Prior to DNA isolation, the strain *A. pullulans* NRRL 62042 (ARS culture collection, Peoria, Illinois, USA) was grown in YPD medium for 20 h at 30 °C and 200 rpm. Genomic DNA was isolated using the Monarch Genomic DNA Purification Kit (New England Biolabs, Frankfurt am Main, Germany). Cells were lysed mechanically according to the protocol provided by NEB using a bead-beater at 6 m s^− 1^ for 40 s (FastPrep-24TM, MP Biomedicals, Santa Ana, Kalifornien, USA). A standard genomic library was created using unique dual indexing, and paired-end 150 bp whole genome sequencing was carried out on an Illumina NovaSeq 6000 (Eurofins Genomics, Ebersberg, Germany). This resulted in 2,611,482,000 sequenced bases and a genome coverage of 104x. A total of 17,409,870 raw reads were cleaned and filtered to 17,254,440 high-quality reads using fastp software for quality control processing [25]. For error correction and normalization, bbnorm was used [26]. The cleaned and normalized reads were assembled using SPAdes (version 3.15.0) [27], resulting in 53 contigs (largest contig: 1,570,265 bp; N50 = 1,070,283 bp; average coverage depth: 97) and 39 scaffolds (largest scaffold: 1,934,914 bp; N50 = 1,169,448 bp; average coverage depth: 97). The genome size was determined to be 24.2 Mb with a GC content of 50.1%. The quality of the assembly was evaluated using QUAST [28] by mapping the reads back to the assembly, resulting in a genome mapping of 99.98%. Additionally, a BUSCO analysis of the assembly was performed [29, 30], yielding a score of 97.1%. Genome annotation for *A. pullulans* 62042 was performed as described recently [31–33] using Genemark v4.68 [34] and GenDBE [35]. For automatic annotation within the platform, similarity searches against different databases, including COG [36], KEGG [37], and SWISS-PROT [38] were performed. In addition to genes, putative tRNA genes were identified with tRNAscan-SE [39]. In total, 9,596 genes were annotated. A BUSCO analysis of the predicted genes [29, 30] resulted in a score of 97.8%. Data sets 1 and 2 (Table 1) were deposited in the NCBI Bioproject PRJNA972899 [40], Biosample SAMN35102111 [41].

**Table 1:** Overview of data files/data sets

Label	Name of data file/data set	File types (file extension)	Data repository and identifier (DOI or accession number)
Data set 1	AP_62042.custom.alignment	Binary Alignment Map (.bam)	NCBI Sequence Read Archive [http://identifiers.org/ncbiprotein:SRR27493674] [42]
Data set 2	AP62042sub	Fasta (.fa)	NCBI GenBank Database [http://identifiers.org/ncbiprotein:JASGXD010000000] [43]

## Limitations

The data presented in this genome note is limited to a single draft genome sequence. Additionally, the genome sequence was generated by Illumina sequencing and is thus relatively fragmented. To overcome these limitations, the data provided here should be set in the context of other sequenced *Aureobasidium* genomes. Furthermore, long-read sequencing methods like Nanopore or Pacbio sequencing could be used to generate a completely assembled genome.

## Data Availability

The data described in this data note can be freely and openly accessed on NCBI Bioproject PRJNA972899 and Biosample SAMN35102111. The Whole Genome Shotgun project has been deposited at GenBank under the accession JASGXD000000000. The version described in this paper is version JASGXD010000000. Please see Table 1 for details and links to the data.

## Abbreviations

bp: Base pairs
